# Some aspects of trypsin’s involvement in the regulation of physiological functions

**DOI:** 10.3389/fmolb.2025.1750770

**Published:** 2026-01-06

**Authors:** I. V. Kuzmina, S. M. Tolpygo, V. G. Vertiprakhov

**Affiliations:** 1 Federal Research Center for Innovator and Emerging Biomedical and Pharmaceutical Technologies, Moscow, Russia; 2 Timiryazev Russian State Agrarian University—Moscow Agrarian Academy, Moscow, Russia

**Keywords:** gastrointestinal tract, poultry, proteinase-activated receptors, rats, trypsin

## Introduction

Currently, the relevance of studying serine proteases secreted by exocrine glands into the bloodstream is increasing due to the increased interest in research related to protease-activated receptors (PARs). PARs are a family of G protein-coupled receptors consisting of four members and characterized by an irreversible proteolytic activation mechanism (Chandrabalan and Ramachandran, 2021). PARs are localized on the cell membranes of various organs and tissues, and their functional activity is regulated by interaction with various proteases such as trypsin, thrombin, etc (Zhao et al., 2014). Currently, serine proteases are considered not only as classical enzymes, but also as signaling molecules actively involved in the regulation of physiological and pathological processes not only in the gastrointestinal tract, but also in other body systems (Aleynik et al., 2018).

Previously, such a serine protease as trypsin was considered as one of the main digestive enzymes that specifically cleave the peptide bonds of proteins.

It is generally recognized that trypsin is an agonist of PAR2 receptors. It has been shown that trypsin, by activating PAR-2, which are present in large quantities on the surface of acinar cells and pancreatic duct cells, enhances cytokine secretion and regulates the exocrine function of the pancreas due to the negative feedback mechanism (Hirota et al., 2006).

## Opinion

### The involvement of trypsin in the regulation of physiological functions in animals of various species

There is evidence of the specific involvement of trypsin in the regulation of physiological functions in animals of various species.

For example, in experiments on rats, it was found that restricting access to water led to a significant increase in trypsin activity in the blood, and restricting feed caused a decrease in this indicator compared with animals with free access to food and water (Kuzmina et al., 2023). Studies on Leghorn roosters have shown that after feeding, there was an increase in trypsin activity in the blood and a multiple increase in the content of nitric oxide donors, which was confirmed by an increase in the concentration of Fe(NO)n in plasma (Vertiprakhov et al., 2023a).

There is evidence that PAR2 is expressed on both vascular endothelium and smooth muscle cells, suggesting its role in the regulation of vascular reactivity. The effects of the activating peptide PAR2 (PAR2-AP) were inhibited by L-NAME, which indicates the involvement of nitric oxide (NO) in these processes. It was shown that the direct effect of trypsin on vascular smooth muscle cells PAR2 caused venoconstriction. It should be noted that arterial vasodilation initiated by the action of vascular PAR2 agonists promoted the release of vasodilating mediators such as NO (Alberelli and De Candia, 2014). It is known that NO is involved in a number of physiological processes, including vasodilation, platelet function regulation, neurotransmission, immunomodulation, and control of metabolic processes (Jon, 2022). The relationship between the NO level and trypsin activity was also discovered during experiments on chickens (Vertiprakhov and Ovchinnikova, 2022).

It was found that an imbalance between the content of NO and endothelins may presumably indicate the development of necrotizing pancreatitis. However, the role of trypsin in the development of this imbalance remains unclear. It is known that the regulation of NO formation in the body is largely related to the renin-angiotensin system (RAS). It was revealed that there is a tissue race in the pancreas and all its main components present in the pancreas are activated when it is damaged. Moreover, in this pathology, trypsin can cause the formation of angiotensin II (AngII) from angiotensinogen at a slightly acidic pH, regardless of the action of the angiotensin-converting enzyme (Furukawa et al., 2013). It has also been established that local RAS is involved in the production of NO in the intestinal mucosa. Stimulation of AT2 receptors (AT2R) AngII of the pig jejunum mucosa led to an increase in the release of NO by epithelial cells into the intestinal lumen (Zamolodchikova et al., 2016).

In the regulation of hemodynamics and vascular tone, the mechanism of NO release is important, which can be induced by AngII processing products that have pronounced vasodilating properties. These metabolites play a key role in the modulation of vascular reactivity and can significantly influence the processes of blood pressure regulation (Dielis et al., 2005). It has been demonstrated that trypsin, when administered intramuscularly, causes a decrease in blood pressure and an increase in heart rate in rabbits (Galiga et al., 2025). This is consistent with data on the hypotensive effect of PAR2 activation (Chia et al., 2011; Wang et al., 2022).

It was noted that in cows, goats and poultry, trypsin activity in blood serum varies in various ways. When analyzing the enzyme activity in the blood of laying hens, a positive relationship was found between egg production and trypsin activity, which makes it possible to use this indicator to assess metabolic processes in the bird’s body (Vertiprakhov et al., 2023b). In this regard, it is interesting to consider trypsin as a PAR2 agonist in the gastrointestinal tract.

### Trypsin, an agonist of PAR2, in the regulation of the gastrointestinal tract

Digestive proteases such as trypsin and chymotrypsin are usually synthesized as inactive precursors. Pancreatic acinar cells express several isoforms of trypsinogen: trypsinogen I, II, IV and mesotrypsinogen. After secretion from the pancreatic ducts, trypsinogen is converted into trypsin on the brush border of enterocytes under the action of enteropeptidase. Active trypsin, in turn, promotes its own activation, acts on chymotrypsinogen, activates PAR-2, thereby increasing paracellular permeability (Agawa et al., 2022). It should be noted that the gastrointestinal epithelium is a promising area of PAR research due to the fact that the gastrointestinal tract is constantly exposed to the greatest number of proteases, both luminal and secreted by the mucous membrane.

A number of studies have revealed that PAR is expressed throughout the gastrointestinal tract, from the salivary glands to the stomach, along the entire length of the intestine, in the pancreas and liver (Hyun et al., 2010; Oliveira et al., 2021).

It has been established that PAR2 modulates acute and chronic inflammatory processes in the gastrointestinal tract in both humans and animals (Lambertini et al., 2020; Zhao et al., 2012).

The intestine is an effective barrier that protects the body from the penetration of pathogenic microorganisms and potentially harmful macromolecules, and also performs its main function—digestion and absorption of nutrients (Weström et al., 2020). The intestine is also one of the main components of the immune system, designed to protect the body from harmful microbes and toxins that enter it with animal feed (Kulkarni et al., 2021).

It has been found that increased intestinal proteolysis and signaling via PAR2 are associated with inflammatory bowel diseases and irritable bowel syndrome, conditions that are often associated with changes in the intestinal microbiome (Lakemeyer et al., 2025).

Special consideration should be given to such a group of pathogenic factors as mycotoxins, which are often present in the feed of farm animals and birds.

Mycotoxins are harmful secondary metabolites produced by mold fungi and have a detrimental effect on human and animal health (Eskola et al., 2020). There are more than 200 known species of molds that produce mycotoxins. The most common harmful mycotoxins polluting feed and feed materials include aflatoxin B1 (AFB1), HT-2, T-2, ochratoxin A, fumonisin B1, zearalenone, citrinin, etc. (Binder, 2007). T-2 and HT-2 toxins are often found in poultry feed, which are among the most dangerous trichothecene mycotoxins and cause gastroenteritis, necrosis of the skin and oral mucosa, as well as disorders of the central nervous system (Gómez-Osorio et al., 2024).

The results of studies on broiler chickens with a 12-finger cannula have shown that experimental mycotoxicosis caused by T-2 toxin results in a decrease in trypsin activity in blood plasma. In freeze-dried droppings with mycotoxicosis, on the contrary, there was a 2.0-fold increase in trypsin activity compared with the control (Vertipra et al., 2022).

Considering the role of trypsin as a PAR2 agonist in the intestine, as well as the variety of functions performed by PARs in this organ, and their wide presence in various cell types, it can be concluded that this enzyme is important in the development of gastrointestinal pathology caused by mycotoxins (Soreide et al., 2006).

The functional role of the intestinal microbiota and its contribution to the transmission of PAR signals through various microbial proteases or by stimulating the activity of proteases in the intestinal mucosa, as well as the differential regulation of PAR expression profiles in the intestine, requires further study (Gummadi and Gonska, 2025).

## Conclusion

These data are consistent with the idea that trypsin is not only a digestive enzyme, but also an important multifunctional signaling molecule that, as a PAR2 agonist, plays an important role in regulating a number of metabolic processes.

The results of numerous experimental studies reviewed in this article suggest that the assessment of trypsin activity in blood serum can serve as a diagnostic method for detecting inflammatory processes and exocrine insufficiency of the pancreas, as well as inflammatory processes in the intestine. Therefore, trypsin can be considered as an informative biomarker of the state of the animal body.
